# Sanitary and environmental conditions related to food poisoning among informal street vendors in downtown Medellín, Colombia, 2016

**DOI:** 10.47626/1679-4435-2023-806

**Published:** 2023-04-18

**Authors:** María Osley Garzón Duque, Fabio León Rodríguez Ospina, Luisa María Saldarriaga Callejas, Juan José Amaya Alviar, Doris Cardona Arango, Maríana Ochoa Sanchez, Ángela María Segura Cardona, María Camila Tamayo Mejía, Iordana Mejía Kambourova

**Affiliations:** 1 Facultad de Medicina, Universidad CES, Medellín, Antioquia, Colombia; 2 Facultad Nacional de Salud Pública, Universidad de Antioquia, Medellín, Antioquia, Colombia; 3 Escuela de Graduados, Facultad de Medicina, Universidad CES, Medellín, Antioquia, Colombia

**Keywords:** informal workers, food supply, sanitary profiles, working environment, occupational risks, trabajadores informales, abastecimiento de alimentos, perfiles sanitarios, ambiente de trabajo, riesgos laborales

## Abstract

**Introduction:**

Although studies with informal workers have been conducted in Latin America and the Caribbean, there is still scarce evidence on the prevalence of food poisoning among workers with subsistence jobs on the streets and sidewalks of cities and on the factors that influence its presentation.

**Objectives:**

To determine sociodemographic, labor, sanitary, and environmental conditions that influence the prevalence of food poisoning in informal workers in downtown Medellín, Colombia.

**Methods:**

This is a cross-sectional study using a workers’ survey as the primary source. A total of 686 workers aged ≥18 years who had been working for ≥ 5 years were surveyed. An assisted survey was initially applied as a pilot test for training purposes and to obtain informed consent.

**Results:**

We identified several associations and explanatory factors of food poisoning using chi-square tests and prevalence ratios, with unadjusted and adjusted 95% confidence intervals (95%CI). A higher prevalence of food poisoning (p < 0.05) was observed in workers with lower frequency of waste collection (PR = 2.09; 95%CI = 1.04-4.19), who left cooked food (PR = 6.40; 95%CI = 2.34-17.8), beverages or chopped fruits uncovered at their workplace (PR = 3.92; 95%CI = 1.40-10.48), with inadequate waste management (PR = 4.84; 95%CI = 2.12-11.06), exposure to polluted water (PR = 3.00; 95%CI = 1.20-7.50) and acceptable water supply (PR = 5.40; 95%CI = 1.60-17.8). The factors that explained higher rates of food poisoning were not having a waste collection service (PR_adjusted_ = 5.58; 95%CI = 3.38-13.10), inadequate waste management (PR_adjusted_ = 6.61; 95%CI = 1.25-34.84), and having sanitary services next to worker’s stall (PR_adjusted_ = 14.44; 95%CI = 1.26-165.11).

**Conclusions:**

The conditions that are associated with and explain the higher prevalence of food poisoning in this working population can be addressed with health promotion and disease prevention interventions.

## Abstract (es)

**Introducción:**

Aunque se han realizado estudios con trabajadores informales en América Latina y el Caribe, aun es escasa la evidencia con respecto a la prevalencia de intoxicaciones alimentarias en trabajadores con empleos de subsistencia en las calles y aceras de las ciudades y a los factores que influyen en su presentación.

**Objetivos:**

Determinar las condiciones sociodemográficas, laborales, sanitarias y ambientales que influyen en la prevalencia de intoxicación alimentaria en trabajadores informales del centro de Medellín.

**Métodos:**

Estudio transversal con fuente primaria de información en un censo de 686 trabajadores, ≥18 años y con ≥5 años en su oficio. Se aplicó encuesta asistida previa realización de prueba piloto, estandarización de encuestadores y toma de consentimiento.

**Resultados:**

Se identificaron asociaciones y factores explicativos de intoxicación alimentaria, con pruebas del chi-cuadrado y razones de prevalencia (RP), con intervalos de confianza del 95% (IC95%) crudos y ajustados. Mayor prevalencia de intoxicación alimentaria (p < 0,05) en trabajadores con menor frecuencia de recolección de residuos (RP = 2,09; IC95% = 1,04-4,19), en quienes dejaban alimentos preparados (RP = 6,40; IC95% = 2,34-17,8), bebidas o fruta picada descubiertos en el puesto de trabajo (RP = 3,92; IC95% = 1,40-10,48), con inadecuado manejo de residuos (RP = 4,84; IC95% = 2,12-11,06), exposición a agua contaminada (RP = 3,00; IC95% = 1,20-7,50) y de aceptable calidad (RP = 5,40; IC95% = 1,60-17,8). Explicaron mayor intoxicación alimentaria no tener servicio de recolección de residuos (RP_ajustada_ = 5,58; IC95% = 3,38-13,10), inadecuado manejo de residuos (RP_ajustada_ = 6,61; IC95% = 1,25-34,84), y tener servicios sanitarios al lado del puesto de trabajo (RP_ajustada_ = 14,44; IC95% = 1,26-165,11).

**Conclusiones:**

Las condiciones que se asocian y explican mayor prevalencia de intoxicación alimentaria en esta población trabajadora, pueden ser revertidas con acciones de promoción de la salud y prevención de la enfermedad.

## INTRODUCTION

Urban growth and pace of changes in the urban environment led that, over the last decades, informal trading on streets and sidewalks become an important economic activity in Latin America and the Caribbean.^1-3^

Street food vending, in turn, is an issue of public health interest worldwide, as shown by Velásquez Gamarra et al.^4^ in their study on the health quality of sandwiches sold in the city of Huacho, México, at formal and informal restaurants and kiosks, which identified that the microorganisms that developed in greater numbers were total coliforms (60%). The authors also report that kiosks and carts do not have drinking water supply nor appropriate containers for waste; moreover, retailers of these establishments good manufacturing practices.^4^

In Colombia, the Epidemiological Bulletin of the Public Health Surveillance System (*Sistema de Vigilancia en Salud Pública,* SIVIGILA) for week 52 of 2018 identified foodborne diseases as an important public health problem, due to the increase in its occurrence, the development of new forms of transmission, and the emergence of vulnerable population groups.^5^

In the week 51 of 2018, 881 outbreaks were reported, whereas there were 859 outbreaks for the same week of 2017, and 668 for the same week of 2016. For the year 2018, the department of Antioquia was found to be a high-risk area, with 35 outbreaks, which accounted for 55.6% of the cases.^5^ However, these reports do not include outbreaks specifically involving informal workers and even less informal street vendors, who sell their products on streets and sidewalks and who can simultaneously sell and consume street food products. This situation reveals a knowledge gap and the need for generating information that help improve life and health conditions of subsistence workers,^6^ who also have a low educational level and low incomes, which makes it difficult for them to have safe working conditions,^7^ such as access to drinking water service, refrigeration, and food cooking, in the case of those who sell products on the streets, being some of the important conditions that should be evaluated to determine the quality of products and their infectious potential for consumers and for vendors themselves.^8,9^

Considering that food poisoning (FP) is a syndrome caused by the consumption of products contaminated with microorganisms or their toxins that generate a systemic response with gastrointestinal symptoms such as nausea, vomiting, diarrhea, abdominal pain, and sometimes fever, representing a worldwide public health problem,^10^ as well as an issue of interest for subsistence workers.

Although some studies^4,11-14^ show that food vendors can be a potential source of contagion for those who buy from them, there is scarce evidence from studies assessing how labor, sanitary and environmental conditions of informal street workers are related to foodborne diseases (FP) experienced by workers, who can simultaneously be sellers and buyers of street food.

For the reasons given above, the present study aimed to determine the labor, health, environmental conditions and the services of the point of sale and its surroundings that are related to FP among informal street vendors in downtown Medellín, Colombia, in 2016, in order to provide evidence that can be included in policies and health promotion programs in the workplace, so as to improve life and health conditions of these workers, considering that a health promotion program in the workplace has already been conducted with workers of Corabastos market in Bogotá, Colombia.^15^

## METHODS

Cross-sectional study with primary sources of information, derived from a doctoral thesis approved by the Institutional Human Ethics Committee - Universidad CES, record No. 84 - code 470 of 2015.

### POPULATION

A survey was conducted with 686 informal street vendors in downtown Medellín who sold goods and pots, seasonable and perishable products, fast food, beverages, appetizers, and desserts, and were contacted by their leaders and by the primary investigator at their market stalls and at guild assemblies. An assisted survey was administered at one of workers’ guild headquarters, after informed consent was obtained from each worker. The study and all its activities were arranged with workers’ leaders and with workers themselves. The study included individuals aged ≥18 years who had been working in downtown Medellín ≥5 years and who agreed to participate after being informed about the study, its procedures, benefits, scopes, and limitations. No participant was excluded according to the established criteria.

### VARIABLES

Dependent variable: prevalence of self-reported FP (due to food consumption during their working hours (1. Yes; 2. No), over the 6 months prior to the survey. Data were collected from February to July 2016, after training sessions with the primary investigator and a public health professional who supported data collection.

Independent variables:

- Sociodemographic conditions: age, sex, and marital status.
- Labor conditions: daily working hours, weekly working days, seniority in the job and in the informal street vending sector (in years), type of vendor, type of product.
- Sanitary workplace conditions: access to public services, sewage, pipeline, public
- lighting, clean and tidy workplace, deteriorated floors and walls, use of personal protection equipment, location and use of sanitary service, type of solid waste disposal.
- Risk factors for the onset of plagues at worker’s market stall: leaving cooked food and beverages or chopped fruits uncovered; inappropriate waste management at worker’s market stall, point of sale near contaminated area or site, type of pollution generated by the area or site, time that the worker remain near the polluted area, quality of water supply. Workers’ household food insecurity was also assessed using the Latin American and Caribbean Food Security Scale (*Escala Latinoamericana y Caribeña de Inseguridad Alimentaria y Nutricional*, ELCSA).^16^

### INSTRUMENT

A questionnaire was specifically designed for the study with the participation of workers and specialists on the theme and subject to format and content validation. A pilot test was conducted before data collection to train the investigators with regard to use of the instrument and data collection in general. No instrument adjustment was necessary after the pilot test. Selection biases were controlled by taking the workers by census, information using an instrument validated in form and content with thematic experts, with the workers and their leaders, standardizing the researchers and a fieldwork assistant and the participants with a process of approach, awareness and explanation of the study, its risks, benefits and potential uses of the information.

### ANALYSIS

Bivariate and multivariate descriptive analyses were performed to explore non-causal associations between independent variables and self-report of poisoning related to food consumed during workers’ working hours. The chi-square test of statistical association was performed, and prevalence ratios (PR) with 95% confidence interval (95%CI) were calculated to establish the strength of association between presence of FP and the previously described independent variables. Variables that contributed to explain the occurrence of FP in workers were evaluated using multiple logistic regression with explanatory purposes, including variables that presented p-values < 0.25 in the bivariate analysis, according to Hosmer-Lemeshow criteria. Analysis was performed using the Epidat software, version 3.1, developed by the Galician Health Service (Servizio Gallego de Saúde) in Galicia, Spain.

## RESULTS

### SOCIODEMOGRAPHIC AND LABOR CONDITIONS AND FOOD INSECURITY

Workers were predominantly male (57.8%), more than a half were single (52.3%), and 64.9% (353) were aged ≥45 years.

More than 80% (364) of participants worked for more than 8 hours a day and 6 and 7 days a week. A half of workers (50.7%) had >20 years of job seniority, and 88.4% (599) had been working >5 years at informal food vending sector. Furthermore, four out of five (80.0%) were semi-stationary or itinerant vendors, and most of them sold goods/pots (59.0%), whereas 41.0% sold seasonable/perishable products, fast food, beverages, appetizers, and desserts (Table 1).

**Table 1:** Sociodemographic and labor conditions of informal street vendors participating in the study. Medellín, 2016

Variable	n	%
Sociodemographic conditions		
Sex		
Male	392	57.8
Female	286	42.2
Marital status		
With a partner	322	47.7
Without a partner	353	52.3
Age		
18 to 44 years	238	35.1
45 years or older	440	64.9
Food insecurity - ELCSA scale		
Moderate to severe	364	53.9
Mild or absent	312	46.1
Labor conditions		
Daily working hours		
More than 8 hours	546	80.5
8 hours or less	132	19.5
Weekly working days		
6 to 7 days	656	96.8
5 days or less	22	3.2
Seniority in the job		
More 20 years	344	50.7
20 years or less	334	49.3
Seniority in the informal street vending sector		
More than 5 years	599	88.4
5 years or less	79	11.6
Type of vendor		
Stationary	121	17.8
Semi-stationary or itinerant	557	82.2
Type of product		
SP, FF, BAD	278	41.0
Goods and pots	400	59.0

BAD = beverages, appetizers, and desserts; FF = fast food; SP = seasonable and perishable products.

The prevalence of moderate/severe household food insecurity in workers was 53.9% (364) (Table 1).

### SERVICES AT WORKER’S MARKET STALL AND ITS SURROUNDINGS

It was found that 36.1% (245) of workers did not have public services at their market stall, with greater rates of absence of electricity supply (12.2%) and of waste collection (8.2%). Moreover, 93.9% (644) considered that their market stall was clean and tidy, 35.5% (244) reported that there were damaged floor or walls at their stall and its surroundings. Only 27.4% (185) used personal protective equipment (Table 2).

**Table 2:** Conditions of worker’s market stall and its surroundings and prevalence of food poisoning in informal street vendors participating in the study. Medellín, 2016

Characteristic	n	%
Conditions of worker’s stall		
Damaged floors and walls		
Yes	241	35.5
No	437	64.5
Public lighting		
Yes	360	53.1
No	318	46.9
Pipeline		
Yes	5	0.7
No	673	99.3
Sewage		
Yes	4	0.6
No	674	99.4
No public services		
Yes	245	36.1
No	433	63.9
Other public services		
Yes	20	2.9
No	658	97.1
Waste disposal		
Temporary storage site		
Yes	35	5.2
No	643	94.8
Collector trolley		
Yes	185	27.3
No	493	72.7
Container		
Yes	226	33.3
No	452	66.7
Informal waste picker		
Yes	41	6.1
No	637	93.9
Other type of final waste disposal		
Yes	293	43.2
No	385	56.8
Risk factors for the development of plagues at worker’s market stall		
Dirty and untidy immediate surroundings		
Yes	288	42.5
No	389	57.5
Accumulated waste or biodegradable material in its surroundings		
Yes	222	32.8
No	255	67.2
Accumulated sewage in its surroundings		
Yes	217	32.1
No	460	67.9
Unprotected grooves or openings		
Yes	22	3.3
No	655	96.7
Cooked food left uncovered		
Yes	4	0.6
No	673	99.4
Beverages or chopped fruits left uncovered		
Yes	10	1.5
No	667	98.5
Inadequate waste management		
Yes	11	1.6
No	666	98.4
Other risk factor		
Yes	150	22.2
No	527	77.8
No risk factor		
Yes	167	24.7
No	510	75.3
Type of pollution generated by the polluted area or site		
Noise		
Yes	528	87.4
No	76	12.6
Unpleasant odors		
Yes	301	49.8
No	303	50.2
Visual pollution		
Yes	62	10.3
No	542	89.7
Air pollution		
Yes	543	89.9
No	61	10.1
Water pollution		
Yes	19	3.2
No	585	96.8
Other type of pollution		
Yes	17	2.8
No	587	97.2
Clean and tidy stall		
Yes	637	93.9
No	41	6.1
Use of personal protective equipment		
Yes	185	27.4
No	489	72.6
Use of sanitary services		
From a neighboring site		
Yes	402	59.3
No	276	40.7
Public restroom		
Yes	258	38.1
No	420	61.9
Pouch or jar		
Yes	5	0.7
No	673	99.3
Other type of sanitary service		
Yes	35	5.2
No	643	94.8
Location of sanitary service		
Next to workplace	29	4.3
Near workplace	531	78.3
Block next to workplace	86	12.7
Several blocks far from workplace	24	3.5
No sanitary service	8	1.2
Frequency of waste collection		
No established frequency	26	3.8
Three times a day	227	33.5
Twice a day	298	44.0
Once a day	116	17.1
Other	11	1.6
Stall or workstation near polluted site		
Yes	604	89.2
No	73	10.8
Waste accumulation in open spaces		
Yes	215	35.6
No	389	64.4
Waste accumulation in stationary waste buckets		
Yes	63	10.4
No	541	89.6
Waste accumulation in containers provided by EEVV		
Yes	10	1.7
No	594	98.3
Sources of residual water (sewage)		
Yes	207	34.3
No	397	65.7
Area of night clubs, bars, and ice-cream shops		
Yes	10	1.7
No	594	98.3
Marketplace		
Yes	23	3.8
No	581	96.2
Vehicle fleet		
Yes	485	80.3
No	119	19.7
Restaurant		
Yes	17	2.8
No	587	97.2
Other (meat and food sales, forkalift drivers, street dwellers, town criers, addiction consumers, parking lots, commercial premises, excrements, construction sites etc.)		
Yes	348	57.6
No	256	42.4
Time near polluted area		
From 1 to 5 hours a day	45	7.5
From 6 to 10 hours a day	392	64.9
More than 10 hours a day	167	27.6
Quality of water supply		
Very good	354	52.5
Good	269	40.0
Acceptable	6	0.9
Fair	26	3.9
Poor and very poor	7	1.0
Do not drink water	12	1.7
Electricity service		
Yes	83	12.2
No	595	87.8
Waste collection		
Yes	56	8.2
No	622	91.8
Food poisoning		
Yes	54	8.0
No	624	92.0

EEVV = Empresas Varias de Medellín: autonomous organization of the City of Medellín, Colombia, responding for delivering cleaning services, understood as waste collection (especially solid waste) and supplemental activities of transportation, sweeping, and cleaning of public routes and spaces, treatment, utilization, and final disposal of waste, lawn mowing, and trimming of trees in public routes and spaces.

### SOCIODEMOGRAPHIC AND LABOR CONDITIONS ASSOCIATED WITH FOOD POISONING IN INFORMAL WORKERS

Most informal workers (94.8%) had no temporary site to store solid waste, nor collector cart (72.7%), nor a container to store waste (66.7%), and only 6.1% (42) delivered waste to an informal picker. However, 44.0% (302) informed that waste was collected twice a day (44%) (Table 2).

With regard to sanitary services, nearly 60.0% of workers reported using a sanitary service at a neighboring site, and 38.1% (258) used the public restroom. It is worth highlighting that five workers said that they used a pouch or a jar as an alternative type of sanitary service. Nevertheless, most participants (78.3%) stated that the sanitary service they used was near their market stall (Table 2).

When exploring factors that could favor the development of plagues or rodents at worker’s market stall, > 42.0% of workers reported its surroundings were dirty an untidy, and a significant percentage said that there was accumulated waste or biodegradable material (32.8%) and accumulated sewage (32.1%) at their market stall (Table 2).

Additionally, 89.2% (604) of vendors reported working near a polluted area or site, and 64.9% (445) told that they remained near this area from 6 to 10 hours a day. Polluted areas or sites were mainly related to waste accumulation in open spaces (35.6%) or in stationary waste buckets (10.4%). Furthermore, 34.3% of workers (235) reported that their market stall was near residual water, and more than 80.0% considered that source of pollution was automotive fleet (Table 2).

The main types of pollution to which workers considered to be exposed were noise (87.4%) and air pollution (89.9%). However, almost a half (49.8%) reported being exposed to unpleasant odors (49.8%) and, in a smaller proportion, to visual pollution or water pollution (Table 2).

Finally, with regard to self-report of FA over the last 6 months prior to data collection, the prevalence of FP in these workers was 7.96% (54) (Table 2).

Although no statistically significant association was observed between sociodemographic and labor conditions and prevalence of FP, the prevalence of this condition was higher in workers without a partner (PR = 1.32), aged from 18 to 44 years (PR = 1.60), with ≥20 years of seniority in the job (PR = 1.21), and >5 years working in the informal food vending sector (PR = 1.29). Women showed a 27.0% higher prevalence of FP compared to men (PR = 0.73) (Table 3).

**Table 3:** Sociodemographic and labor conditions associated with food poisoning in informal street vendors participating in the study. Medellín, 2016

Characteristic	Food poisoning		Total	Chi-square test (p-value)	PR (95%CI)
	Yes n (%)	No n (%)			
Sociodemographic conditions					
Marital status					
Without a partner	32 (9.0)	321 (91.0)	353	1.14 (0.285)	1.32 (0.78-2.23)
With a partner	22 (6.8)	300 (93.2)	322		1.0
Sex					
Male	27 (6.9)	365 (93.1)	392	1.470 (0.225)	0.73 (0.44-1.22)
Female	27 (9.4)	259 (90.6)	286		1.0
Age					
18 to 44 years	25 (10.5)	213 (89.5)	238	3.22 (0.070)	1.60 (0.95-2.65)
45 or older	29 (6.6)	411 (93.4)	440		1.0
Labor conditions					
Daily working hours					
More than 8 hours	44 (8.1)	502 (91.9)	546	0.03 (0.850)	1.06 (0.549-2.057)
8 hours or less	10 (7.6)	122 (92.4)	132		1.0
Weekly working days					
6-7 days	52 (7.9)	604 (91.1)	656	0.04 (0.840)	0.87 (0.23-3.35)
5 days or less	2 (9.1)	20 (90.9)	22		1.0
Seniority in the profession					
More than 20 years	30 (8.7)	314 (91.3)	344	0.54 (0.460)	1.21 (0.72-2.03)
20 years or less	24 (7.2)	310 (92.8)	334		1.0
Seniority in the vending sector					
More than 5 years	49 (8.2)	550 (91.8)	599	0.32 (0.560)	1.29 (0.53-3.14)
5 years or less	5 (6.3)	74 (93.7)	79		1.0
Type of vendor					
Stationary	10 (8.3)	111 (91.7)	121	0.02 (0.890)	1.05 (0.54-2.02)
Semi-stationary - itinerant	44 (7.9)	513 (92.1)	557		1.0
Type of product					
Goods and pots	31 (7.8)	369 (92.3)	400	0.06 (0.800)	0.94 (0.56-1.57)
SP, FF, BAD^**^	23 (8.3)	255 (91.7)	278		1.0

Values in bold font: statistically significant association when p < 0.05.

95%CI = 95% confidence interval; BPD = beverages, appetizers, and desserts; FF = fast food; PR = prevalence ratio; SP = seasonable and perishable products.

### SANITARY AND ENVIRONMENTAL CONDITIONS OF THE WORKPLACE AND ITS SURROUNDINGS ASSOCIATED WITH FOOD POISONING IN INFORMAL WORKERS

Statistically significant associations (p < 0.05) were observed between higher prevalence of FP and presence of waste collection service, frequency of collection, leaving cooked food, beverages, and chopped fruits unexposed at the workplace, inappropriate management of solid waste at worker’s market stall, environment pollution, and quality of water supply (Table 4).

**Table 4:** Services and sanitary and environmental conditions of worker’s market stall and its surroundings associated with food poisoning in informal street workers participating in the study. Medellín, 2016

Characteristic	Food poisoning		Total	Chi-square test (p-value)	PR (95%CI)
	Yes n (%)	No n (%)			
Services of worker’s market stall and its surroundings					
Public lighting					
Yes	30 (8.3)	330 (91.7)	360	0.14 (0.700)	1.10 (0.66-1.85)
No	24 (7.5)	294 (92.5)	318		1.0
Pipeline					
Yes	1 (20)	4 (80)	5	0,02 (0,860)	1,0
No	53 (7,9)	620 (92,1)	673		2.54 (0.43-14.94)
Use of pouch or jar as an alternative sanitary service					
Yes	1 (20.0)	4 (80.0)	5	0.02 (0.860)	2.54 (0.43-14.94)
No	53 (7.9)	620 (92.1)	673		1.0
Use of sanitary service in public restrooms					
Yes	21 (8.1)	237 (91.4)	258	0.01 (0.890)	1.03 (0.61-1.75)
No	33 (7.9)	387 (92.1)	420		1.0
Use of sanitary service in a neighboring site					
Yes	33 (8.2)	369 (91.8)	402	0.08 (0.770)	1.08 (0.64-1.80)
No	21 (7.6)	255 (92.4)	276		1.0
No public service					
Yes	16 (6.5)	229 (93.5)	245	1.07 (0.300)	0.74 (0.42-1.31)
No	38 (8.8)	395 (91.2)	433		1.0
Waste collection service					
Yes	17 (30.4)	39 (69.6)	56	41.75 (0.000)	5.10 (3.08-8.45)
No	37 (5.9)	585 (94.1)	622		1.0
Location of sanitary services					
Next to worker’s stall	6 (20.7)	23 (79.3)	29	11.11 (0.02)	2.48 (0.55-11.2)
Near worker’s stall	34 (6.4)	497 (93.6)	531		0.76 (0.20-3.01)
Next block	11 (12.8)	75 (87.2)	86		1.53 (0.36-6.45)
No service	1 (12.5)	7 (87.5)	8		1.50 (0.15-14.4)
Several blocks far from worker’s stall	2 (8.3)	22 (91.7)	24		1.0
Frequency of waste collection					
No established frequency	1 (3.8)	25 (96.2)	26	5.4 (0.140)	0.62 (0.08-4.50)
Twice a day	24 (8.1)	274 (91.9)	298		1.30 (0.69-2.46)
Once a day	15 (12.9)	101 (87.1)	116		2.09 (1.04-4.19)
Three times a day	14 (6.2)	213 (93.8)	227		1.0
Dirty and untidy immediate surroundings					
Yes	25 (8.7)	263 (91.3)	288	0.33 (0.560)	1.16 (0.69-1.9)
No	29 (7.5)	360 (92.5)	389		1.0
Accumulation of waste or biodegradable material					
Yes	18 (8.1)	204 (91.9)	222	0.007 (0.920)	1.02 (0.59-1.7)
No	36 (7.9)	419 (92.1)	455		1.0
Accumulation of sewages					
Yes	16 (7.4)	201 (92.6)	217	0.15 (0.69)	0.89 (0.50-1.56)
No	38 (8.3)	422 (91.7)	460		1.0
Cooked food left uncovered					
Yes	2 (50.0)	2 (50.0)	4	4.7 (0.02)	6.4 (2.34-17.80)
No	52 (7.7)	621 (92.3)	673		1.0
Beverages or chopped fruits left uncovered					
Yes	3 (30.0)	7 (70.0)	10	4.0 (0.04)	3.92 (1.40-10.48)
No	51 (7.6)	616 (92.4)	667		1.0
Inadequate waste management at worker’s market stall					
Yes	4 (36.4)	7 (63.6)	11	8.6 (0.003)	4.84 (2.12-11.06)
No	50 (7.5)	616 (92.5)	666		1.0
No polluting focus					
Yes	14 (8.4)	153 (91.6)	167	0.05 (0.82)	1.06 (0.60-1.90)
No	40 (7.8)	470 (92.2)	510		1.0
Market stall or workstation near polluted site					
Yes	45 (7.5)	559 (92.5)	604	2.11 (0.14)	0.60 (0.3-1.18)
No	9 (12.3)	64 (87.7)	73		1.0
Time of exposure to pollution					
1 to 5 hours a day	6 (13.3)	39 (86.7)	45	5.9 (0.05)	1.0
6 to 10 hours a day	22 (5.6)	370 (94.4)	392		0.40 (0.18-0.98)
More than 10 hours a day	17 (10.2)	150 (89.8)	167		0.70 (0.30-1.80)
Polluting source generated water pollution					
Si	4 (21.1)	15 (78.9)	19	3.4 (0.06)	3.00 (1.20-7.50)
No	41 (7.0)	544 (93.0)	585		1.0
Polluting source generated unpleasant odors					
Si	20 (6.6)	281 (93.4)	301	0.50 (0.40)	0.80 (0.40-1.40)
No	25 (8.3)	278 (95.7)	303		1.0
Quality of water supply					
Very good	22 (6.2)	332 (93.8)	354	8.4 (0.08)	1.0
Good	26 (9.7)	243 (90.3)	269		1.50 (0.90-2.70)
Acceptable	2 (33.3)	4 (66.7)	6		5.40 (1.60-17.8)
Fair	3 (11.5)	23 (88.5)	26		1.80 (0.60-5.80)
Poor/very poor	1 (14.3)	6 (85.7)	7		2.30 (0.30-14.70)
Market stall with damaged floors or walls					
Si	21 (8.7)	220 (91.3)	241	0.28 (0.59)	1.15 (0.68-1.95)
No	33 (7.6)	404 (92.4)	437		1.0
Clean and tidy market stall					
Yes	44 (6.9)	593 (93.1)	637	16.06 (0.000)	0.28 (0.15-0.52)
No	10 (24.4)	31 (75.6)	41		1.0
Use of personal protective equipment					
Si	12 (6,5)	173 (93,5)	185	0,54 (0,46)	0,79 (0,42;1,48)
No	40 (8,2)	449 (91,8)	489		1.0

Content in bold font: statistically significant association when p < 0.05.

95%CI = 95% confidence interval; PR = prevalence ratio.

It is worth noting that vendors who did not have a waste collection service showed an 81.0% lower prevalence of FP (PR = 0.19; 95%CI = 0.11-0.32) than those who had this service. In turn, the prevalence of FP was twice as high in workers who had their waste collected once a day compared to those who had it collected three times a day (PR = 2.09; 95%CI = 1.04-4.19) (Table 4).

Conversely, for each worker who did not leave cooked food uncovered at their stall and experienced FP, there were 6.4 workers who left it uncovered and reported having experienced FP (PR = 6.4; 95%CI = 2.34-17.8). Similarly, those who reported leaving beverages or chopped fruits uncovered at their stall had a 2.92-fold higher prevalence of FP compared to those who did not have this habit (PR = 3.92; 95%CI = 1.4-10.48) (Table 4).

Furthermore, a four-fold higher prevalence of FP was found in workers who reported inadequate management of solid waste at their stall (PR = 4.84; 95%CI = 2.12-11.06). Those who reported acceptable water supply showed a 4.4-fold higher prevalence of FP compared to those with very good water supply (PR = 5.4; 95%CI = 1.6-17.8). With regard to the closeness of workers’ stall to residual water (sewage), the prevalence of FP was 2.0-fold higher in those exposed to this type of water (PR = 3.0; 95%CI = 1.2-7.5) (Table 4).

Conversely, a statistically significant association (p < 0.05) was observed between lower prevalence of FP and exposure to polluted area or site from 6 to 10 hours a day (PR = 0.40; 95%CI = 0.18-0.98) and clean and tidy stall (PR = 0.28; 95%CI = 0.15-0.52) (Table 4).

There was a non-significant association between higher prevalence of FP and using a pouch or a jar as a sanitary service (PR = 2.54), having a sanitary service next to worker’s stall (PR =2.48) or on the block next to it (PR = 1.53), not having access to sanitary services (PR = 1.50), and considering the quality of water supply as good (PR = 1.50), fair (PR = 1.80) or poor/very poor (PR = 2.30) (Table 4).

### SANITARY AND ENVIRONMENTAL CONDITIONS AND SERVICES OF WORKER’S STALL AND ITS SURROUNDINGS THAT CONTRIBUTE TO EXPLAIN FOOD POISONING IN INFORMAL WORKERS

The following situation served as explanatory variables of FP in informal workers: not having a waste collection service, inadequate waste management, working near a polluting source (polluted area or site), using a sanitary unit near worker’s market stall, and considering it clean and tidy (Table 5).

**Table 5:** Sanitary and environmental conditions and services of worker’s stall and its surroundings that contribute to explain food poisoning in informal street vendors participating in the study. Medellín, 2016

Condition/characteristic - services, sanitary and environmental conditions of workers’ stall and its surroundings	Unadjusted PR	95%CI		Adjusted PR	95%CI	
		LL	UL		LL	UL
Model 1						
Re-categorized age. 45 and older (Rc)^*^						
18 to 44 years	1.60	0.95	2.65	1.68	0.84	3.33
Sex. Women (Rc^*^)						
Men	0.73	0.44	1.21	0.59	0.30	1.17
Waste collection at worker’s stall. Yes (Rc)^*^						
No	0.19	0.11	0.32	5.58	2.38	13.10
Frequency of waste collection. Three times (Rc)^*^						
With no established frequency	0.62	0.08	4.50	0.41	0.04	3.77
Twice a day	1.30	0.69	2.46	1.47	0.65	3.23
Once a day	2.09	1.04	4.19	1.89	0.75	4.80
Inadequate waste management at worker’s market stall. No (Rc)^*^						
Yes	4.84	2.12	11.06	6.61	1.25	34.84
Workstation near a source of pollution. No (Rc)^*^						
Yes	0.60	0.30	1.18	0.18	0.08	0,42
Time of exposure to source of pollution. 1 to 5 hours a day (Rc)^*^						
6 to 10 hours a day	0.40	0.18	0.98	0.47	0.17	1.34
More than 10 hours a day	0.70	0.30	1.80	0.84	0.28	2.55
Location of sanitary service. Several blocks far from worker’s stall (Rc^*^)						
Next to worker’s stall	4.48	0.55	11.20	14.44	1.26	165.11
Near worker’s stall	0.76	0.20	3.01	2.54	0.28	23.42
Block next to worker’s stall	1.53	0.36	6.45	4.17	0.41	42.37
No sanitary service	1.50	0.15	14.40	9.58	0.41	226.04
Model 2						
Cooked food left uncovered at worker’s stall. No (Rc)^*^						
Yes	6.40	2.34	18.80	12.38	0.76	201.41
Beverages and chopped fruits left uncovered at worker’s stall. No (Rc)^*^						
Yes	3.92	1.40	14.48	3.92	0.62	24.90
Source of pollution generates water pollution. No (Rc)^*^						
Yes	3.00	1.20	7.50	1.10	0.27	4.52
Clean and tidy stall. No (Rc)^*^						
Yes	0.28	0.15	0.52	0.26	0.16	0.67
Water supply at worker’s stall. Very good (Rc)^*^						
Buena	1.50	0.90	2.70	1.47	0.74	2.92
Acceptable	5.40	1.60	17.80	5.63	0.60	53.14
Fair	1.80	0.60	5.80	1.82	0.39	8.54
Poor and very poor	2.30	0.30	14.70	1.32	0.16	10.67

(Rc)^*^: Reference category for comparison. Results are presented for the categories to which this category is compared. Content in bold font: statistically significant association when p < 0.05.

95%CI = 95% confidence interval; LL = lower limit; PR = prevalence ratio; UL = upper limit.

No having a waste collection service at worker’s market stall went from being associated with a lower prevalence of FP (PR_unadjusted_ = 0.19; 95%CI = 0.11-0.32) to explaining a 4.58-fold higher prevalence of this condition (PR_adjusted_ = 5.58; 95%CI = 3.38-13.10) when adjusted for the remaining variables included in the analysis (Table 5).

Conversely, inadequate management of solid waste at worker’s stall maintained its statistical significance (p < 0.05); however, when adjusted for the remaining variables in the analysis, it increased its explanatory capacity, going from being associated with a 3.84-fold higher prevalence (PR_unadjusted_ = 4.84; 95%CI = 2.12-11.06) to explaining a 5.61-fold higher prevalence of FP (PR_adjusted_ = 6.61; 95%CI = 1.25-34.84) (Table 5).

Having a sanitary service near worker’s market stall also increased the strength of association and gained explanatory capacity (p < 0.05), going from being associated with a 3.38-fold higher prevalence of FP (PR_unadjusted_ = 4.48) to explaining a 13.44-fold higher prevalence of FP (PR_adjusted_ = 14.44; 95%CI = 1.26-165.11) (Table 5).

Conversely, lower prevalence of FP in informal workers may be explained by considering that their workstation was near a source of pollution and that their workplace was clean and tidy, since the prevalence of FP was 82.0% lower in workers who considered that they workstation was near a source of pollution (PR_adjusted_ = 0.18; 95%CI = 0.08-0.42), and 74.0% lower in those who considered that their workplace was clean and tidy (PR_adjusted_ = 0.26; 95%CI = 0.16-0.67) (Table 5).

Finally, despite not significantly contributing to explain FP, a higher prevalence of this condition was found in vendors who reported that waste collection was performed one (PR_adjusted_ = 1.89) or twice a day (PR_adjusted_ = 1.47), that sanitary services were near their stall (PR_adjusted_ = 2.54) or on the block next to their stall (PR_adjusted_ = 4.17), that they did not have this type of service (PR_adjusted_ = 9.58), that they left cooked food (PR_adjusted_ = 12.38) or beverages and chopped fruits (PR_adjusted_ = 3.92) uncovered at their stall. Considering that the water workers consumed at their point of sale was good (PR_adjusted_ = 1.47), acceptable (PR_adjusted_ = 5.63), fair (PR_adjusted_ = 1.82), poor and very poor (PR_adjusted_ = 1.32) also contributed to explain a higher prevalence of FP (Table 5).

## DISCUSSION

This study assessed a predominantly male and single working population aged above 45 years, conditions similar to those reported in a national study on informal work that assessed 20 departments in Colombia.^17^ Age showed a variability similar to that found in a study with food handlers on the streets in Uberaba, Minas Gerais, Brazil,^18^ probably due to the different characteristics of the study populations, like it was also observed in the characterization of street vendors in Bogotá.^19^

Most vendors worked for > 8 hours a day, in line with results observed in studies conducted by the Ministry of Social Protection in Colombia,^17^ and in a study performed in Bogotá, in which the average number of working hours was 11.08.^20^ These long working hours were associated with workers’ physical and psychological exhaustion, making them more prone to become ill and increasing time of exposure to risk factors, which favors the development of pathological conditions.^21^

With regard to household food insecurity as assessed by the ELCSA scale,^16^ more than a half of workers experienced moderate to severe household food insecurity, a higher percentage than that reported in a study conducted in the city of Medellín, Colombia, which found that 62% of households experienced some degree of food insecurity, mainly mild or moderate.^22^ The regional^23,24^ and international literature^25,26^ shows that this condition is related to malnutrition and with a higher risk of different diseases. If this condition is added to the fact that most workers were semi-stationary or itinerant vendors, that good percentage sold seasonable and perishable products, and the highest prevalence of FP was found in workers who sold food, this would make these workers increasingly more socially and economically vulnerable.

The above situation is plausible, because these workers have an increased likelihood of contamination due to their type of vending. However, it is noteworthy that one of the studies analyzed revealed that exposure to risk factors was higher among those who worked at fixed stalls and at home,^5,27,28^ differently from what was shown in the present study, in which the prevalence of FP was higher in semi-stationary and itinerant vendors.

A higher prevalence of FP was observed in vendors who had a waste collection service; however, comparison of this evidence with that from previous studies is not possible, due to the scarce information published about the theme. A higher prevalence of FP was also found in those who reported a frequency of waste collection of once a day. This could be partially explained because this frequency would make workers exposed to polluting factors that could favor the onset of gastrointestinal conditions for a longer time.

One of the factors associated with FP was leaving food, beverages, and chopped fruits uncovered at worker’s stall, a finding similar to those presented by the SIVIGILA for Colombia,^5^ which show that 18.3% and 15.7% of poisoning outbreaks are related to inadequate food conservation and inadequate food storage, respectively, reinforcing the hypothesis that these factors could increase the risk for poisoning^5^ in the working population. A higher prevalence of FP was also observed in workers who reported inadequate waste management, including inadequate disposal and waste accumulation at worker’s stall, as well as deficient hand washing after handling waste. Other studies found a higher prevalence of contaminating bacteria on workers’ hands when they have inadequate cleaning practices at their stall.^4,8-10^ These factors potentiate the risk for poisoning by bacteria that cause gastrointestinal conditions.^8,10^

With regard of time of workers’ exposure to the polluted area or site (polluting source), it is worth noting that the prevalence of FP was found to be higher among those who exposed themselves from 6 to 10 hours a day, a length of time that coincides with vendor’s working hours, and this time of exposure is likely to be related to a higher prevalence of poisoning.^8,9,13,14^

Water pollution served as an explanatory factor of a higher prevalence of FP, as evidenced in many studies^4,8,9,13,14^ showing that FP is related to consumption of non-drinking water, lack of drinking water at the workplace, and poor water access. The present study observed a higher prevalence of FP in workers who considered that the quality of water supply was acceptable.

Conversely, workers who considered their market stalls as clean and tidy showed a lower prevalence of FP, a situation that was also observed in a study by Rojas Velasco et al.,^20^ who found that one of the labor risk factors for vendors is a dirty and untidy workstation, as well as entrances and floor in poor conditions, with a prevalence of food poisoning of 27%.

Vendors who used personal protective equipment appropriately had a lower prevalence of FP. Different reports found that one of the main deficiencies in street vending of prepared products is not using personal protective equipment, which is a risk factor for communicable diseases, although 61.0% of the working population assessed in this study reported using this equipment. However, observations conducted with other workers^18,20^ revealed that only 10.0% of them made use of personal protective equipment, showing non-compliance with street vending regulations set forth in Resolution 604, dated of 1993.^29^

Finally, a higher prevalence of FP was observed in vendors who had a waste collection service once a day, who left cooked food, beverages, and chopped fruits uncovered, had inappropriate waste management, and considered that the source of pollution affected water quality. The following situation contributed to explain a higher prevalence of FP: absence of waste collection service, inadequate waste management, and sanitary services near worker’s market stall. A lower prevalence of FP was found in vendors who worked near a polluting source and who considered their market stall as clean and tidy. These are plausible conditions, from the sanitary and environmental point of view, although they are difficult to discuss and compare, due to the scarce published evidence about the theme and the type of analysis described in the literature. We suggest conducting studies that establish explanatory associations and relationships between sanitary, environmental, labor conditions, services at worker’s market stalls, and diseases that may result from these conditions.

A limitation of the study was not evaluating FP by means of laboratory tests. Although the prevalence of FP was self-reported, this information may be useful to advance the development and execution of studies that evaluate this disease among this population using laboratory measures and that result in public health measures, such as registration of FP outbreaks as events of public health interest, which includes informal food street vendors

In conclusion, the conditions that are associated and explain a higher prevalence of FP can be addressed with health promotion and disease prevention interventions. These interventions in turn may contribute to improve life and health conditions of the working population, and could be directed to public policies for Medellín street vendors and their families or to health promotion programs for informal workers, especially for those who sell their products as informal vendors on streets and sidewalks of cities. Moreover, it is recommended to consider the inclusion of cases occurring specifically in population of informal street workers into reports of FP outbreaks published by the Health National Institute (Instituto Nacional de Salud), in order to advance in identifying the extent of this public health problem and advancing measures that reflect the reality of the working population.
